# Dataset on optimization and development of a point-of-care glucometer-based SARS-CoV-2 detection assay using aptamers

**DOI:** 10.1016/j.dib.2021.107278

**Published:** 2021-08-12

**Authors:** Naveen K. Singh, Partha Ray, Aaron F. Carlin, Sydney C. Morgan, Celestine Magallanes, Louise C. Laurent, Eliah S. Aronoff-Spencer, Drew A. Hall

**Affiliations:** a Department of Electrical and Computer Engineering, University of California – San Diego, La Jolla, CA 92093, USA; b Division of Surgical Oncology, Department of Surgery, Moores Cancer Center, University of California – San Diego Health, La Jolla, CA 92093, USA; c Division of Infectious Diseases and Global Public Health, Department of Medicine, University of California – San Diego, La Jolla, CA 92093, USA; d Department of Obstetrics, Gynecology, and Reproductive Sciences, University of California – San Diego, La Jolla, CA 92093, USA; e Department of Bioengineering, University of California – San Diego, La Jolla, CA 92093, USA

**Keywords:** Aptamer, Enzyme, Glucometer, SARS-CoV-2, COVID-19, Point-of-care, Population screening

## Abstract

We present supplementary data for the published article, “Hitting the diagnostic sweet spot: Point-of-care SARS-CoV-2 salivary antigen testing with an off-the-shelf glucometer” [1]. The assay described is designed to be performed at home or in a clinic without expensive instrumentation or professional training. SARS-CoV-2 is detected by an aptamer-based assay that targets the Nucleocapsid (N) or Spike (S) antigens. Binding of the N or S protein to their respective aptamer results in the competitive release of a complementary antisense-invertase enzyme complex. The released enzyme then catalyzes the conversion of sucrose to glucose that is measured by an off-the-shelf glucometer. The data presented here describe the optimization of the assay parameters and their contribution to developing this aptamer-based assay to detect SARS-CoV-2. The assay performance was checked in a standard buffer, contrived samples, and patient samples validated with well-established scientific methods. The resulting dataset can be used to further develop glucometer-based assays for diagnosing other communicable and non-communicable diseases.

## Specification Table

**Table:** 

Subject	Biology
Specific subject area	Biosensor, aptamer-based enzyme-linked assay
Type of data	Tables Graph Figures
How data were acquired	The clinical saliva samples (no additives) were collected with informed consent under Institutional Review Board (IRB) approval (UCSD protocol #200477) from symptomatic and asymptomatic COVID-19 patients. These samples were processed for viral RNA extraction using the MagMax Viral/Pathogen Nucleic Acid Isolation Kit (Thermo) and the TaqPath COVID-19 multiplex RT-qPCR assay (Thermo) was performed on the resulting RNA samples. Aptamer-based displacement optimization and assay data were collected with a household glucometer. The collected data were plotted with Origin version 9 software. The electrophoretic mobility shift assay (EMSA) was performed and analyzed with Bio-Rad analysis and imaging system.
Data format	Raw Analyzed
Parameters for data collection	Aliquots of 200 µg tethered magnetic bead complex (either N or S aptamer/antisense-invertase complex) were incubated with 100 µL Dulbecco's potassium phosphate buffer (DPBS) or 2-fold diluted human saliva, spiked with SARS-CoV-2 N or S protein with gentle shaking for 30 min at room temperature (RT) in a 1.5 mL centrifuge tube. Following the capture of the target antigen by the respective aptamer, the antisense-invertase conjugate was released into solution. The supernatant (90 µL) was collected, after separating the intact magnetic bead complexes with a magnet and transferred into another centrifuge tube prefilled with 30 µL of 4× sucrose cocktail buffer (4 M Sucrose, 0.24 M Glucose, 20 mM CaCl_2_, 4 mM MgCl_2,_ and 2 mM EDTA in 0.4 M citrate buffer of pH 5.0). After mixing, the reaction was incubated in a water bath at 60 °C for 30 min. Lastly, 10 µL of the solution was placed on the glucometer test strip, and the readings were recorded with a glucometer. A similar protocol was followed for virus-infected cell-conditioned media and clinical sample processing. The dataset presented was collected from independent experiments performed in triplicate. The post-experimental data analysis includes the observed change in the glucose value [mg/dL] with significant statistical consideration.
Description of data collection	The presence of target viral antigen (SARS-CoV-2 Spike or Nucleocapsid protein) in the sample indirectly correlated with the replaced antisense-invertase conjugate after binding the target to the respective aptamer over the MB complex. The glucose formed after a fixed incubation time was measured with a glucometer. The glucose level was measured with an “Accu-Chek Guide Me” glucometer as per the standard defined procedure. In brief, a 10 µL drop of solution was placed on parafilm, and the glucose strip was assembled with the glucometer and brought in contact with the drop for 5 s. The displayed signal on the glucometer was then recorded.
Data source location	Institution: University of California San Diego City/Town/Region: La Jolla, California Country: United States of America samples/data: UCSD health sciences
Data accessibility	Data is hosted on Mendeley Data at https://data.mendeley.com/datasets/9scn37td2p/draft?a=0f7d8479-0fba-492f-8c49-0bb2c78f7b7c
Related research article	Singh et al., “Hitting the diagnostic sweet spot: Point-of-care SARS-CoV-2 salivary antigen testing with an off-the-shelf glucometer,” https://doi.org/10.1016/j.bios.2021.113111. (https://www.sciencedirect.com/science/article/pii/S0956566321001482)

## Value of the Data

• These data provide researchers with insight into designing a glucometer-based point-of-care aptamer displacement assay with high sensitivity and specificity.
• These data demonstrate how to improve aptamer-target binding through surface density optimization.
• These data demonstrate the improvement in sensitivity and specificity, with a reduction of assay time, compared to 1× PBS, pH 7.3 at room temperature by optimizing the assay parameters.
• The clinical data allows one to assess the sensitivity (true-positive rate) and specificity (1 – false-positive rate) with different cutoff values using the receiver operator characteristic (ROC) curve.
• The data presented here can be used to conduct similar research using the point-of-care glucometer to target other disease biomarkers.
• The data will support similar diagnostic research in resource-limited areas, where basic medical healthcare facilities are unavailable.

## 1 Data Description

More than one hundred million people worldwide rely on a glucometer daily to manage their blood sugar levels, making glucometers the most prevalent piece of diagnostic equipment globally. However, there are significant hurdles in using a glucometer to detect biomarkers at picomolar (pM) levels. Glucometers are designed to measure physiological levels of blood glucose (*i.e.* ∼10–600 mg/dL or ∼0.6–33 mM), and a standard glucometer is designed to utilize 2×10^12^ glucose molecules/sec over a single glucometer strip [2,3]. This is much higher than the average viral load present in nasal/throat, sputum, or saliva specimens (3×10^6^, 7.50×10^5^, and 3.5×10^7^ per mL, respectively) of COVID-19 patients [4,5]. Hence, the proposed assay employs a highly efficient invertase-based amplification procedure to enhance the signal (up to 10^6^-fold). Invertase is an enzyme with a turnover rate of 5000 glucose molecules/s [6], such that sub-nanomolar levels of invertase can convert sucrose into glucose under ambient conditions.

To translate COVID-19 viral antigen-binding events into glucose production, we engineered a novel aptamer-based competitive assay. For this, aptamers against SARS-CoV-2 nucleocapsid (N) [7] and the receptor-binding domain (RBD) of the spike (S) [8] antigen were used to capture the corresponding target antigens. The 5’ biotin-anti-S (or anti-N) protein aptamers were hybridized to a small oligonucleotide (∼15–25 base-pairs antisense) complementary to a portion of the aptamer sequence. The antisense oligonucleotide was conjugated to an invertase enzyme. This complex was then pre-assembled on magnetic beads (MB) to form a magnetic bead complex (MBC). In the presence of the respective target, the anti-N or anti-S aptamer undergoes a conformation change displacing the complementary antisense strand and thus the invertase. The released oligo-invertase was incubated with sucrose, converting it to glucose, thus amplifying the signal. The resulting glucose was measured with a glucometer and is directly proportional to the viral antigen.

**Fig. 1:**
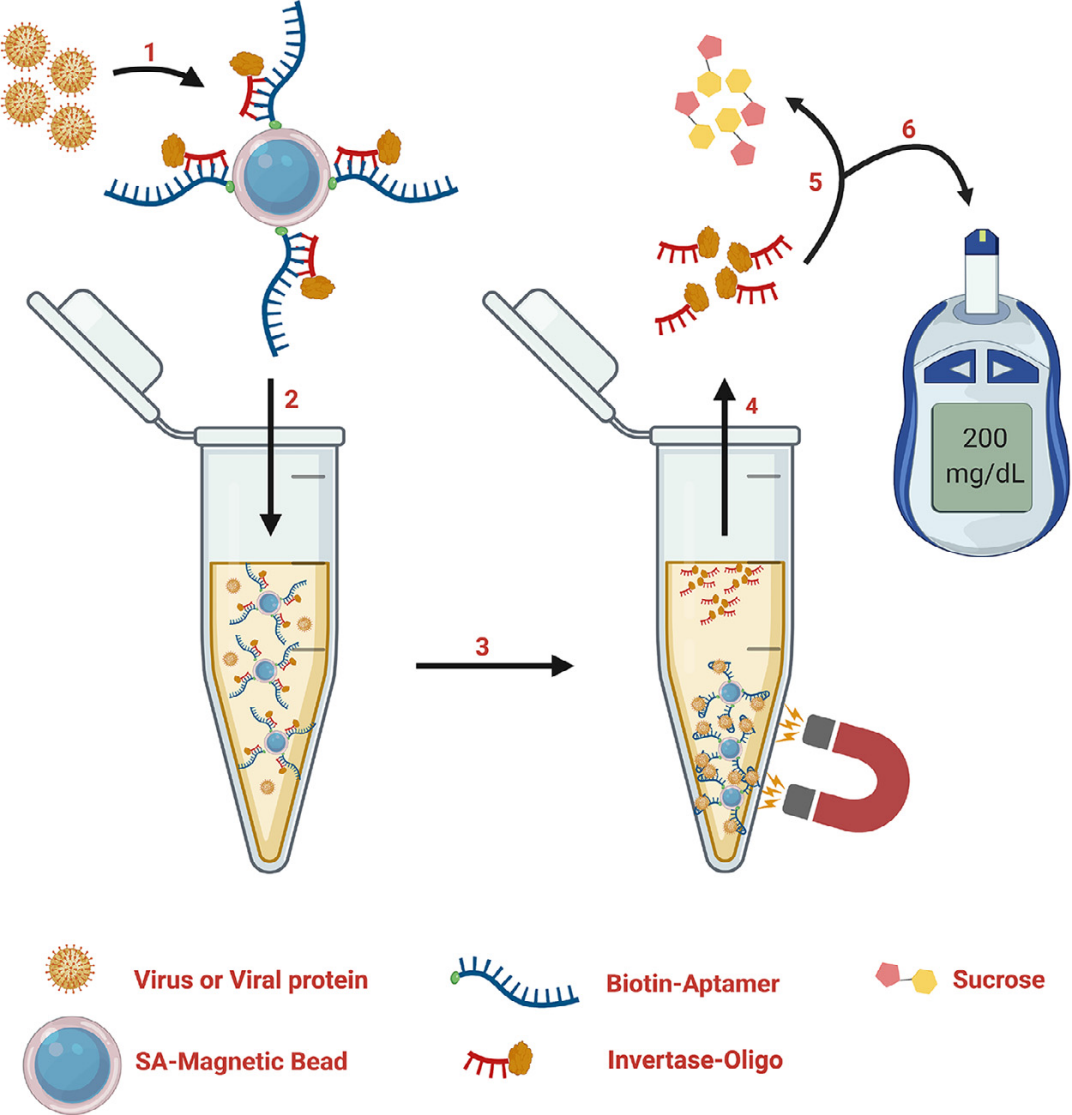
*Assay scheme.* Biotin-aptamer (Anti-S or Anti-N protein) are annealed to the complementary invertase-oligonucleotide and pre-assembled on streptavidin-coated magnetic beads (MB). Next, samples containing SARS-CoV2 virus, or viral (S/N) protein, are incubated with this pre-assembled complex (**Steps 1 and 2**). The binding of the virus or viral protein to the aptamer triggers a conformational switch releasing the invertase-oligonucleotide into solution (**Step 3**). The virus-bound aptamer-MB complexes are separated using a magnet, and the supernatant containing invertase-oligonucleotide is collected (**Step 4**). The invertase-oligonucleotide solution is then incubated with sucrose (**Step 5**), which is converted to glucose and measured by a commercially available glucometer (**Step 6**). This figure was drawn using software from BioRender.com.

**Fig. 2:**
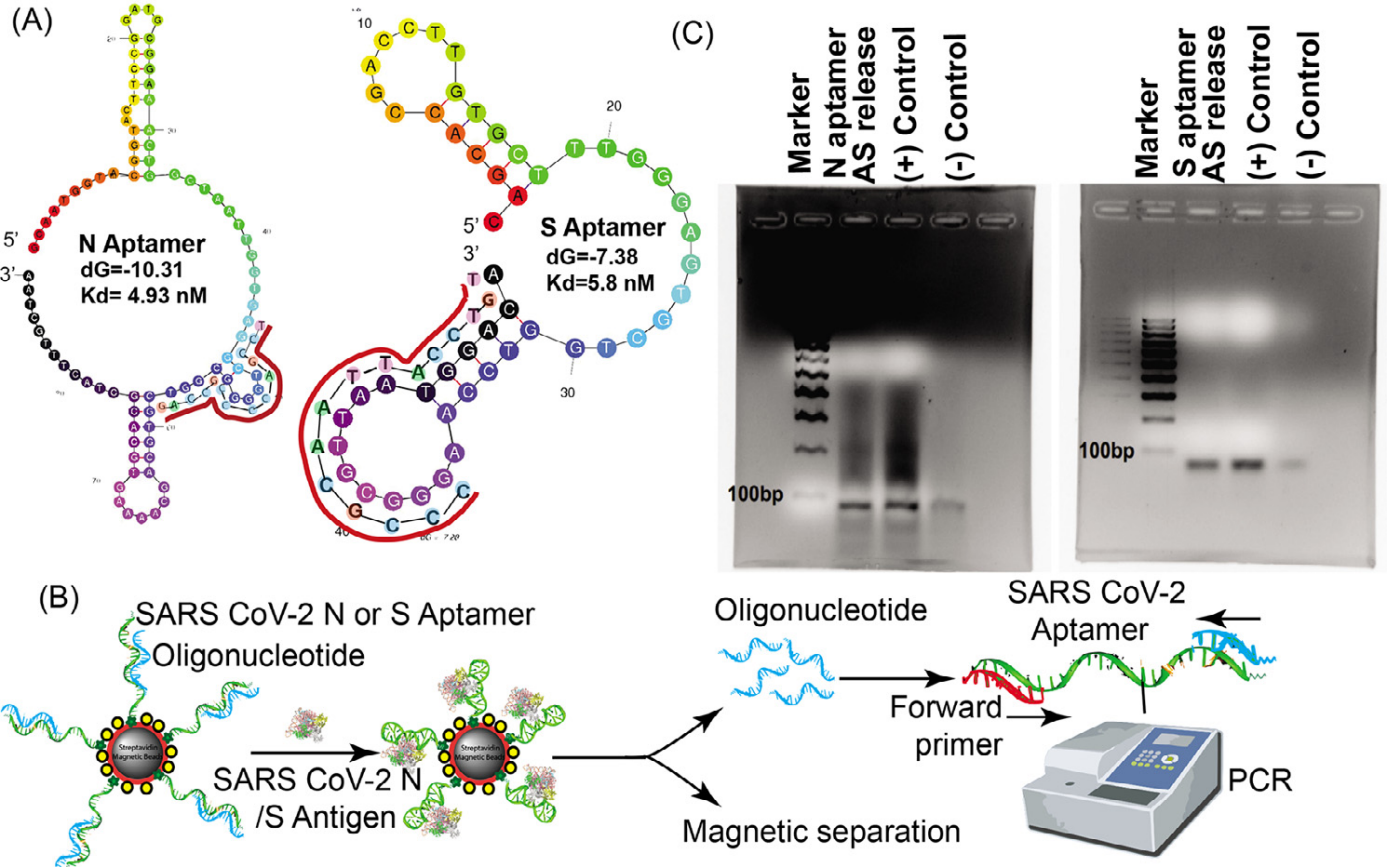
*Aptamer and antisense strand displacement verification.* (A) Predicted secondary structure of the N and S aptamer using M-fold. The antisense strand sequence and the binding locations are annotated in red. (B) Overview of the release study of the antisense strand (blue) from aptamer (green) upon antigen binding and validation study of the release using PCR. After magnetic separation of the MB-aptamer-antigen conjugate, the released oligonucleotide (blue) is collected from the supernatant and is added to the PCR reaction mixture with the aptamer (green) as a template and the forward primer (red). PCR amplification is confirmed by running PCR products on an agarose gel followed by staining with Ethidium Bromide (EtBr). (C) Antisense (AS) release study from the hybridized N and S aptamer immobilized on the magnetic beads upon antigen binding was confirmed with PCR. The PCR products were resolved in 2% agarose gel and stained with EtBr to visualize the DNA amplicon. PCR reactions with the respective aptamer templates, forward and reverse primers were performed for the (+) control. For the (-) control, only buffer (without N or S proteins) was added to the magnetic bead complex (MBC). A 100 base-pair ladder was also resolved as a molecular-weight marker. (For interpretation of the references to color in this figure legend, the reader is referred to the web version of this article.)

**Fig. 3:**
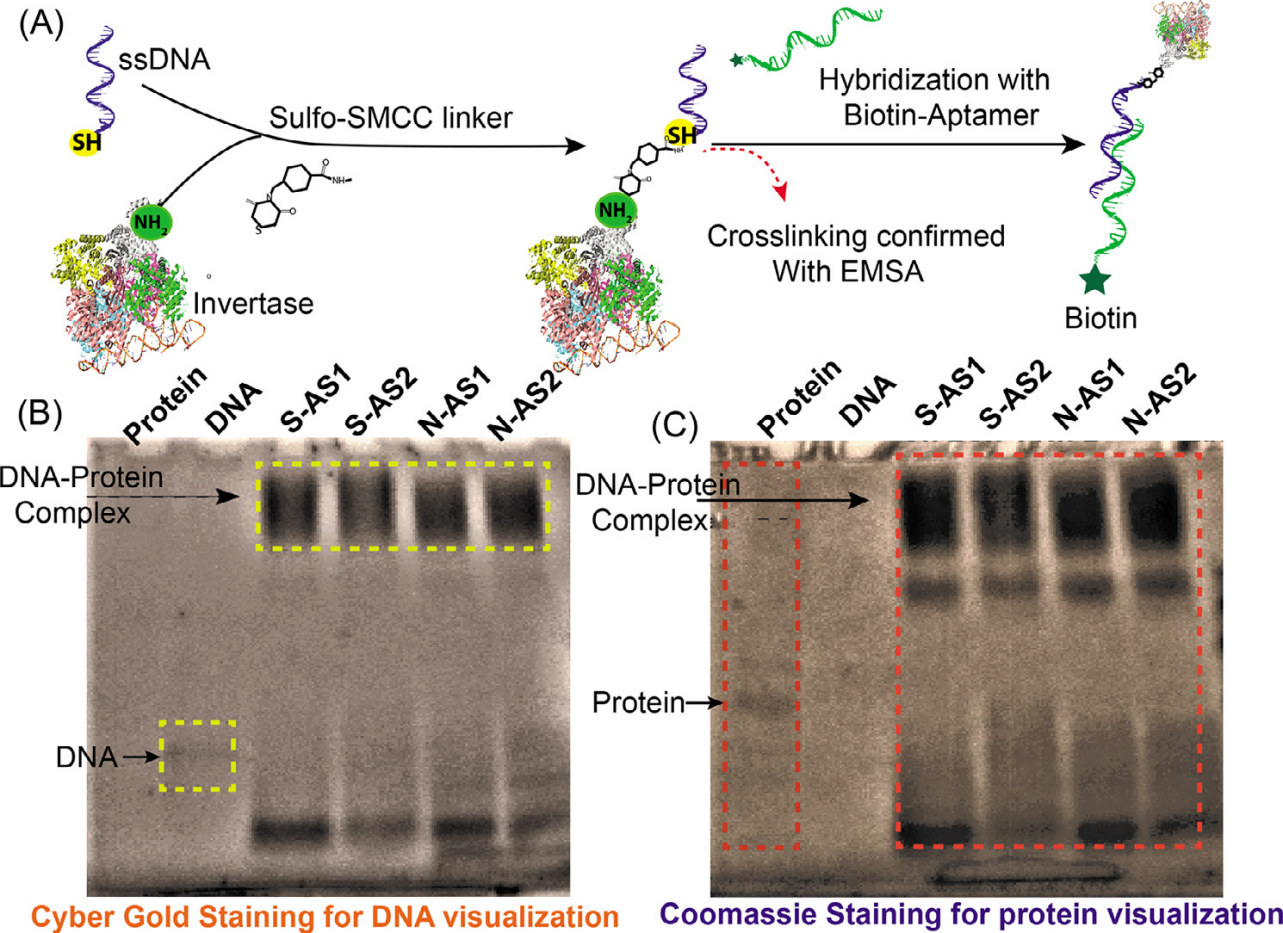
*Conjugation of the antisense-invertase enzyme.* (A) Overview of cross-linking antisense oligonucleotide with invertase and hybridization with the respective biotinylated-aptamer. (B) N-antisense (N-AS) and S-antisense (S-AS) conjugation with invertase was verified by an electrophoretic mobility shift assay (EMSA). The conjugates were resolved in 4–20% gradient native acrylamide gel for 2 h in 1× TBE buffer at 100 V. Unconjugated DNA and the invertase protein were run as controls. S-AS1, S-AS2 and N-AS1, N-AS2 depict two different concentrations of antisense-invertase enzyme conjugates. The gel was stained with (B) Cyber Gold, followed by (C) Coomassie brilliant blue for DNA and protein staining, respectively. Higher migrating bands were detected at the same spot with both the DNA and protein-specific dyes, thus indicating successful conjugation. (For interpretation of the references to color in this figure legend, the reader is referred to the web version of this article.)

**Fig. 4:**
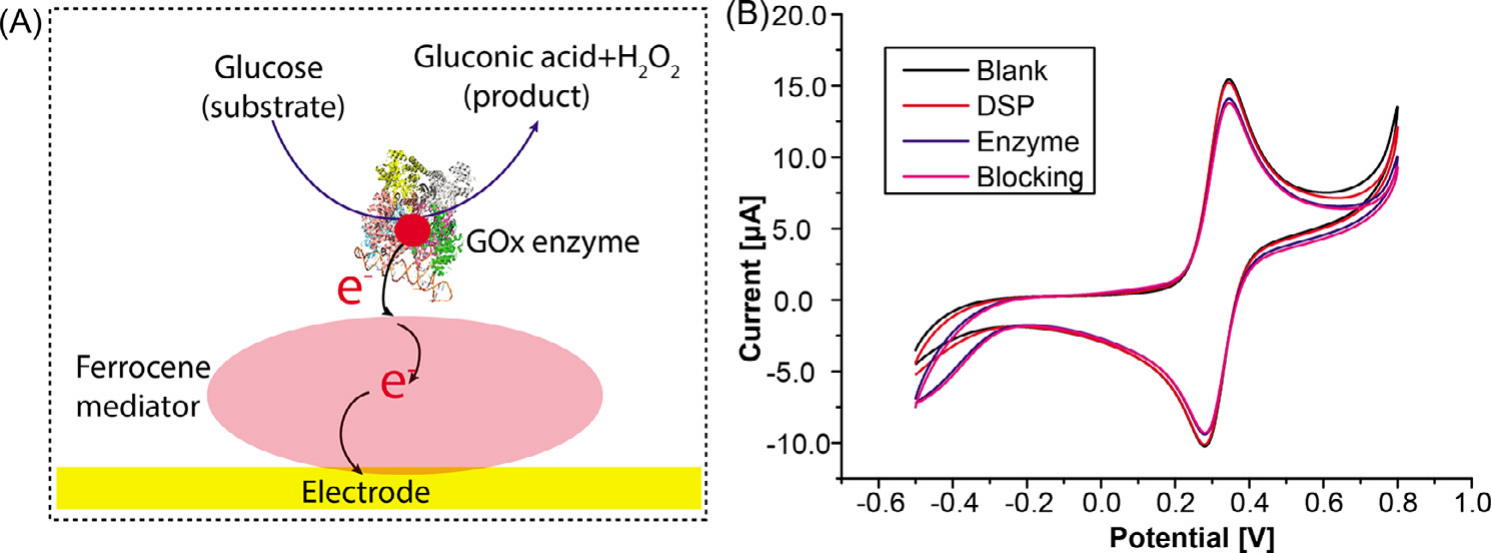
*Custom glucose sensor fabrication and assessment.* (A) Overview of glucose sensor operating principle. (B) Characterization of the sensor fabrication showing a gradual reduction in current after layer-by-layer immobilization of DSP, enzyme (GOx), and blocking confirming successful stepwise assembly.

**Fig. 5:**
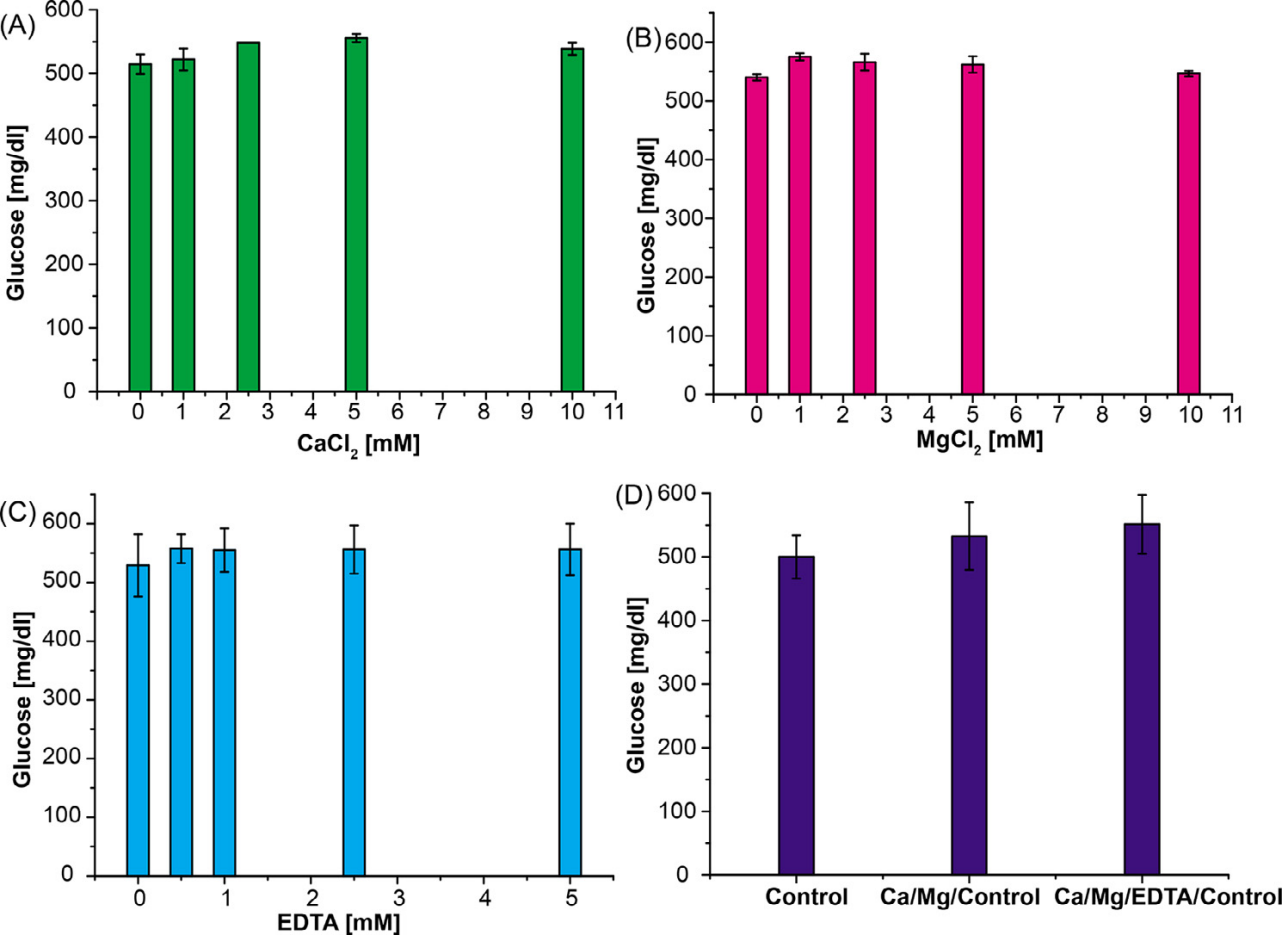
*Ion and salt optimization for amplification buffer.* Metal ions and salts have a crucial role in enzyme activity. An optimization study was performed to test the effects of different concentrations of salts - (A) Ca, (B) Mg, and (C) EDTA - on enzyme activity. The effect of EDTA on metal ion chelation was observed to increase invertase activity without any inhibitory effect. (D) Optimization using best conditions from (A–C). These optimization studies were performed using 1.0 M sucrose with 1 µM invertase enzyme at RT for 30 min. Maximum invertase activity was observed with 5 mM CaCl_2_, 1 mM MgCl_2_, and 0.5 mM EDTA.

**Fig. 6:**
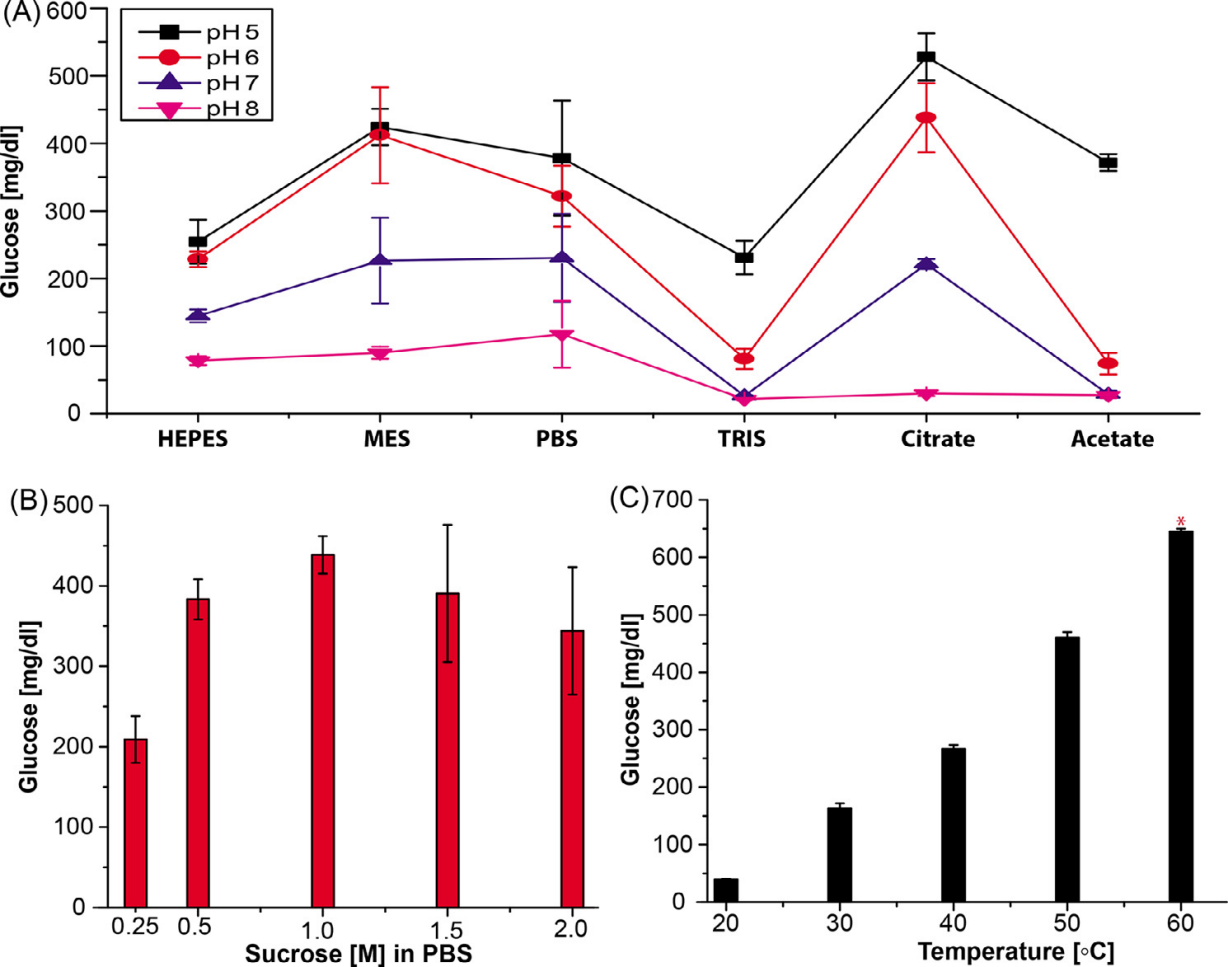
*Invertase enzyme activity and amplification buffer optimization.* (A) Buffer optimization with 0.1 M buffers at different pH. (B) Substrate concentration optimization in 1× PBS. (C) Temperature study in 0.1 M citrate buffer (pH 5.0). *Indicates that the sample was diluted 2-fold due to the limited dynamic range of the glucometer. All the enzyme optimization studies were performed using 1.0 M sucrose with 1 µM invertase enzyme at RT for 30 min. Optimized parameters increase the invertase activity enabling better sensitivity and specificity.

**Fig. 7:**
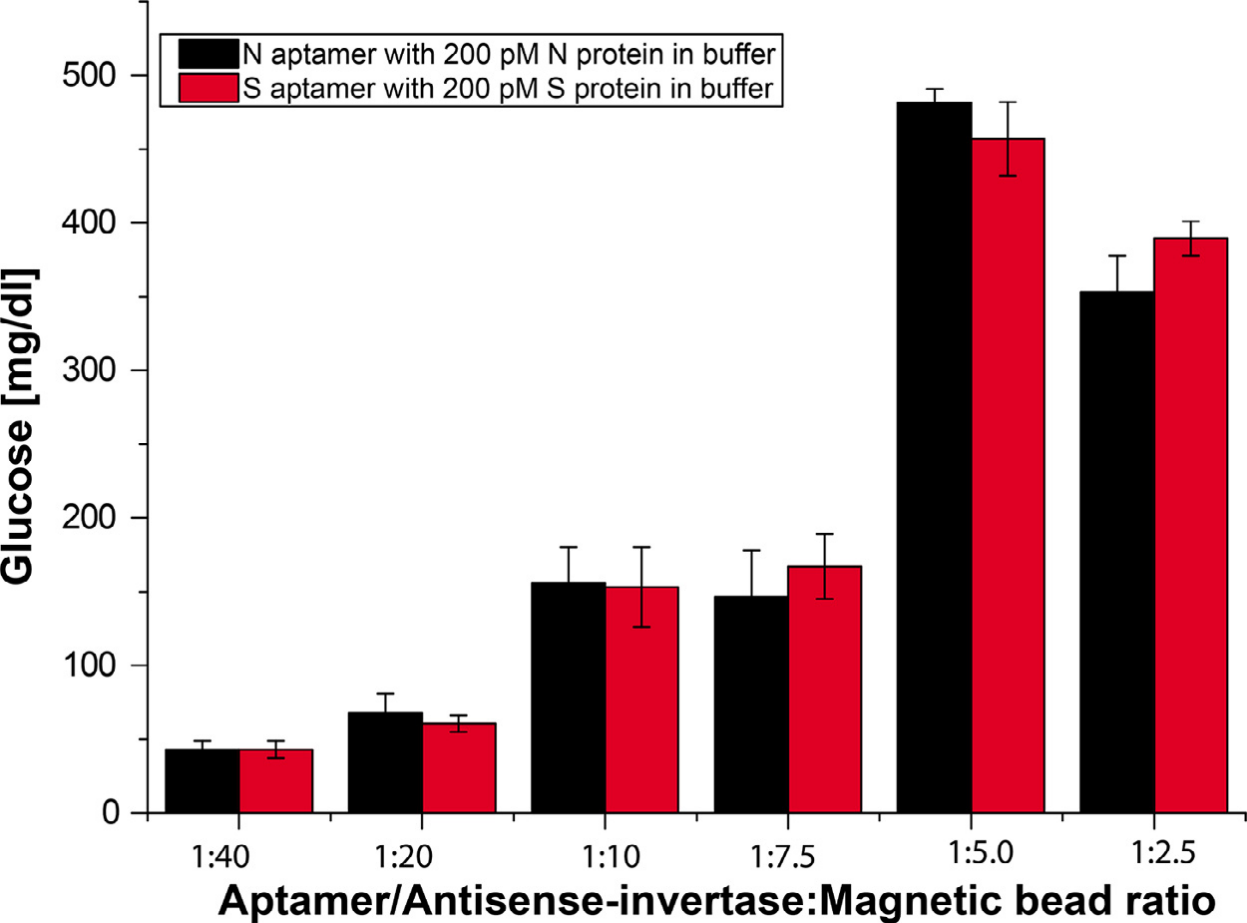
*Effect of the aptamer-antisense-invertase conjugate to streptavidin-coated magnetic bead ratio on antigen binding.* The loading efficiency of the assay system was varied from 1:40 to 1:2.5 ratios and saturated at a 1:5 ratio (60 µg of aptamer to 300 µg of MBs) for both aptamers.

**Fig. 8:**
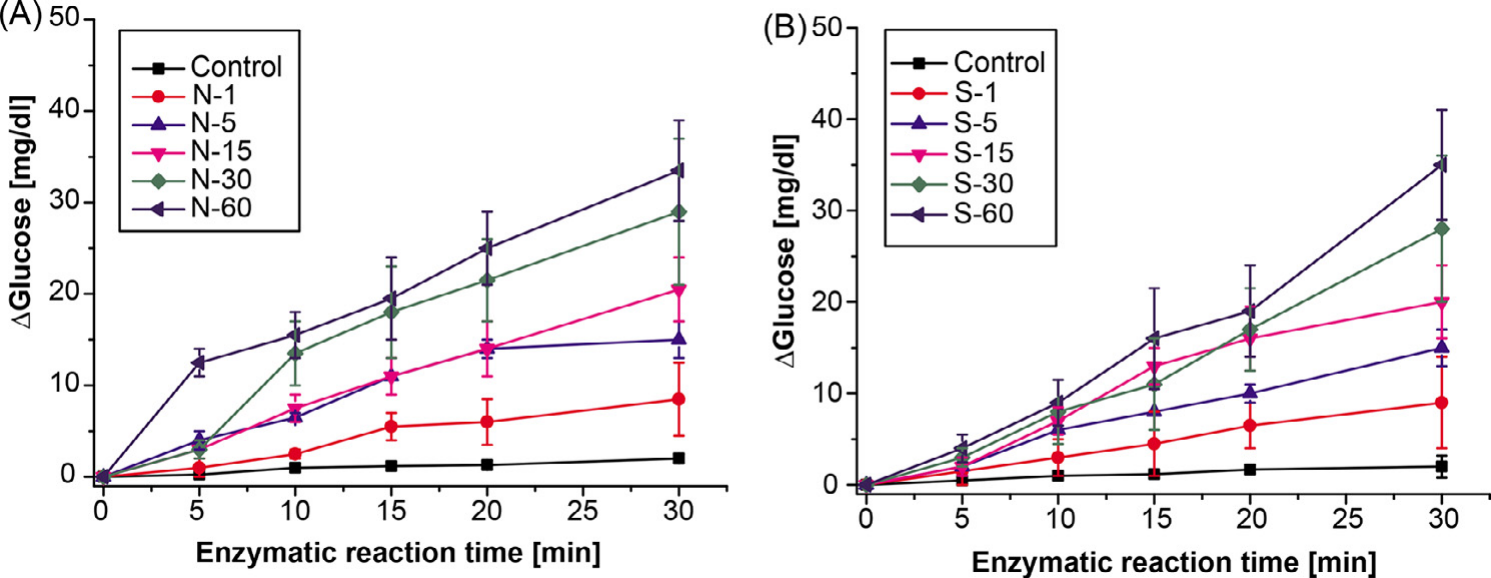
*Enzymatic reaction kinetics.* Aptamer binding and enzymatic reaction time optimization for (A) N and (B) S aptamer complex systems in buffer. N or S aptamer complex was incubated against the respective target for 1 to 60 min, and corresponding invertase activity assay performed up to 30 min at 5 min intervals. The control experiments were performed similarly in the absence of antigen. The assay was performed using the optimal ratio of aptamer/antisense-invertase, as determined in Fig. 6: a 1:5 ratio of MB to N or S aptamer/antisense-invertase-MB complex. As expected, the longer the incubation time for aptamer-target interaction, the higher the concentration of antisense-invertase conjugate released into solution from aptamer/antisense-invertase complex and enhance sucrose conversion rate.

**Fig. 9:**
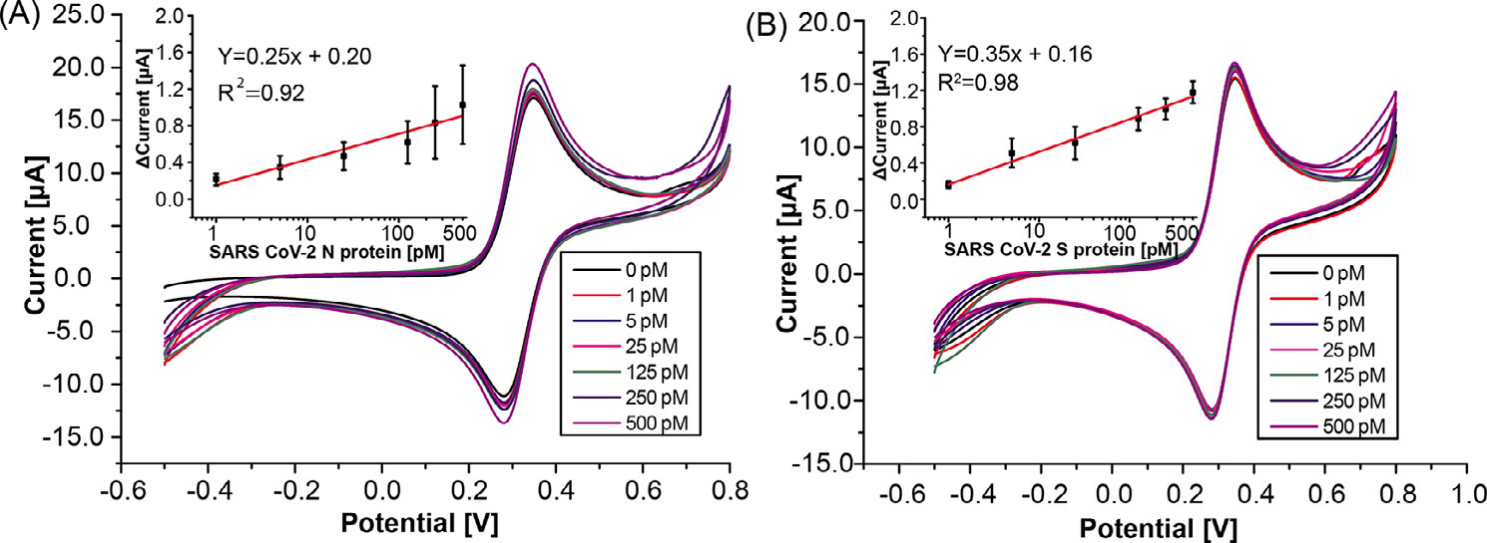
*Measurement of SARS-CoV-2 antigen with the custom glucose sensor.* Measurement results from (A) N and (B) S SARS-CoV-2 protein spiked into a 1× measurement buffer at various concentrations (1–500 pM). Inset shows calibration plot after background subtraction. Measurements were performed in a 1× measurement buffer with a ferrocene mediator to facilitate the electron transfer from the enzyme redox center to the electrode surface. An incremental shift in the oxidation peak of the voltammograms was observed at higher concentrations. The calculated limit of detection (LOD) is 0.71 and 0.34 pM for SARS-CoV-2 N and S protein, respectively. All measurements were performed in triplicate, and error bars represent ±1σ.

**Fig. 10:**
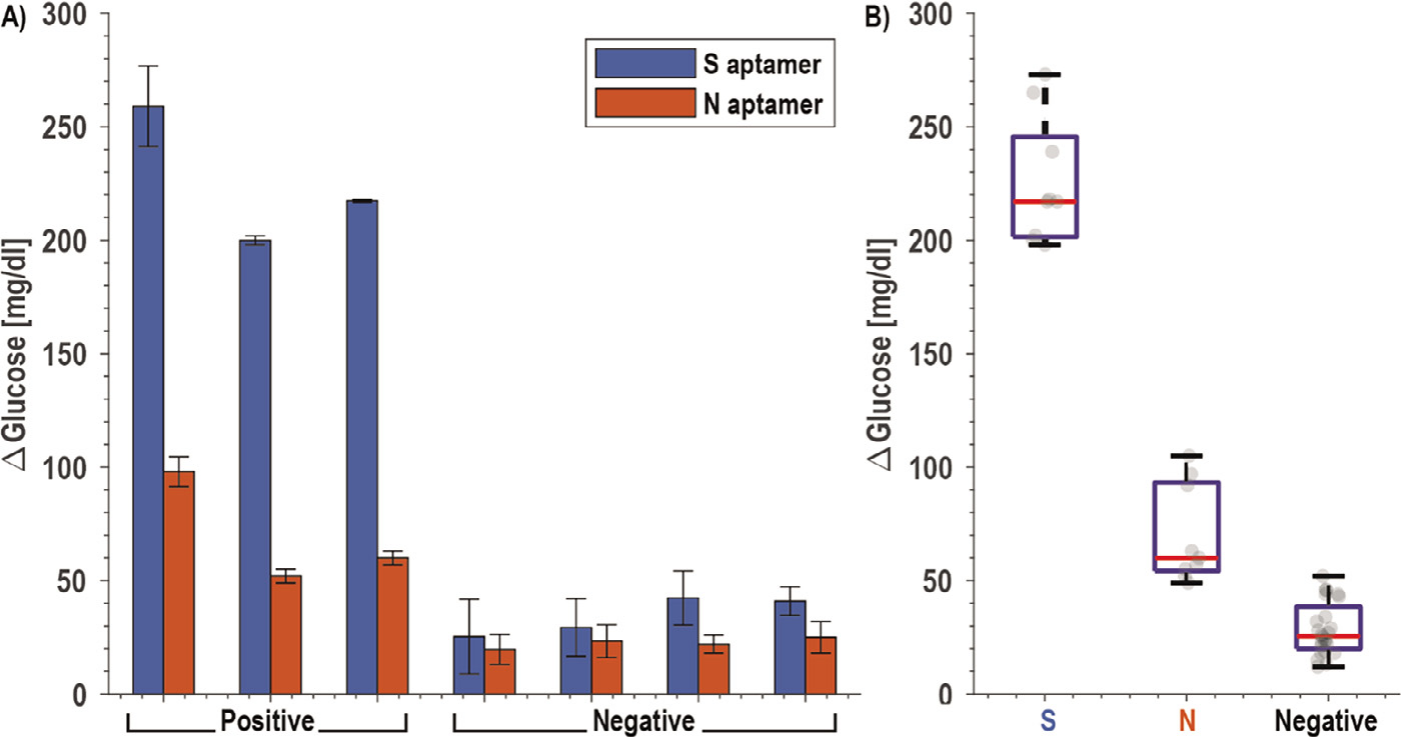
*COVID-19 clinical saliva samples.* (A) Measured data from confirmed positive patients (*n*=3; Patients 23, 30, and 42 [Table 4]) and healthy volunteers (*n*=4) for paired N and S aptamers. Detection of SARS-CoV-2 N protein was performed with 1% Triton to ensure the release of the nucleocapsid protein. (B) Box and whisker plot showing the same data as (A).

**Fig. 11:**
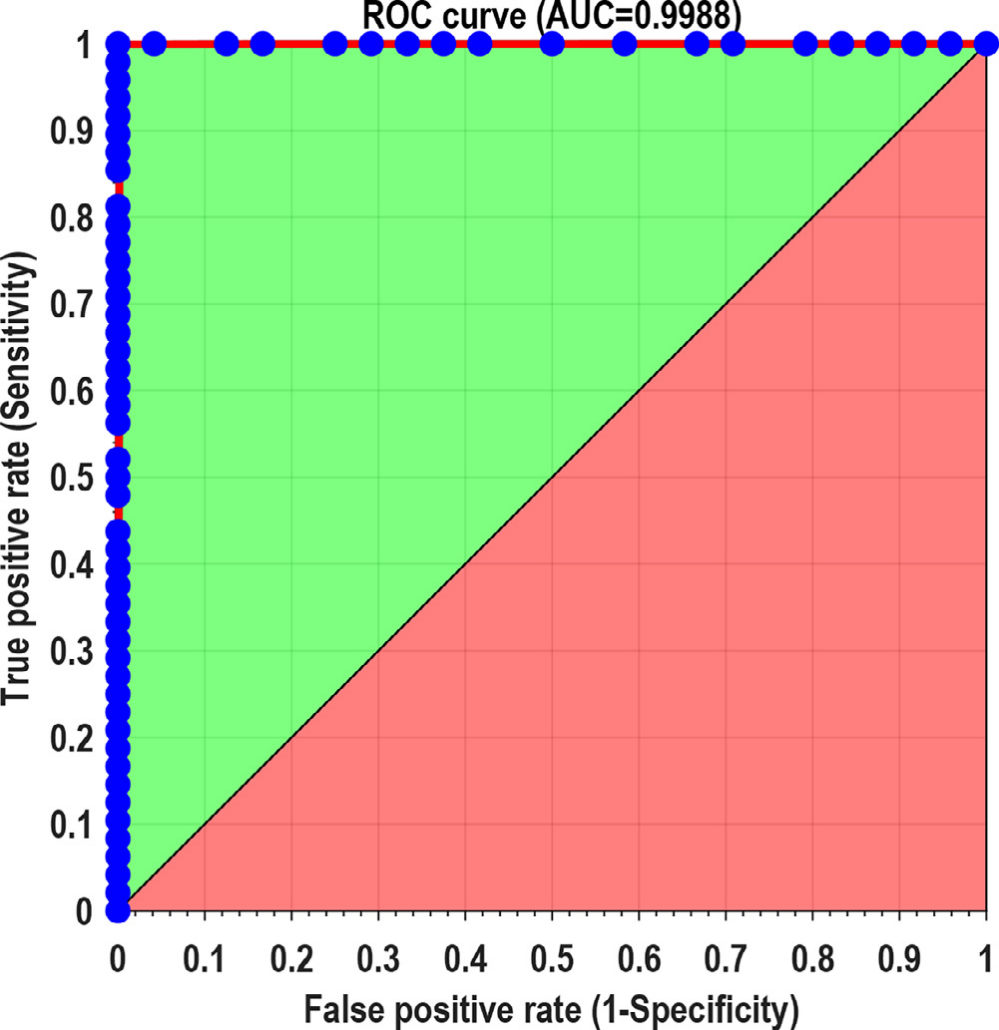
*Receiver operator characteristic (ROC) curve***.** All individual measurements were plotted using MATLAB with the function provided by Giuseppe Cardillo [10]. This curve shows the tradeoff between the sensitivity and specificity for different cutoff values.

**Fig. 12:**
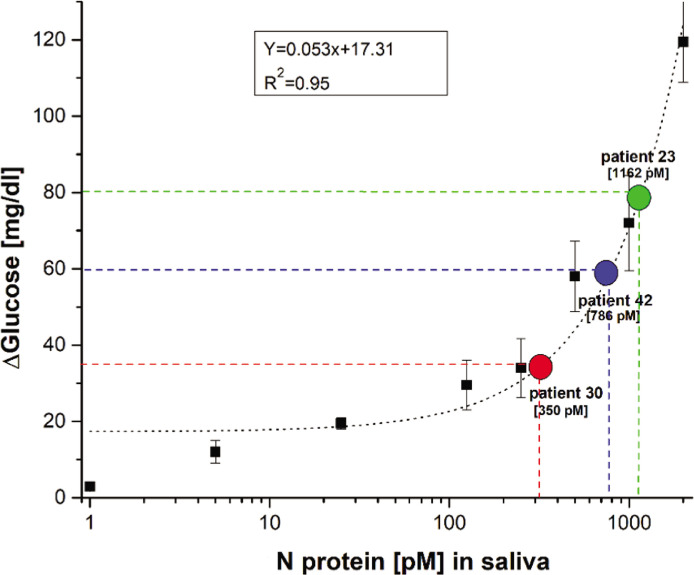
*Estimation of nucleocapsid (N) protein in clinical samples.* The antigen concentration for clinical samples was interpolated based on the calibration curve generated from contrived saliva samples.

Schematic representation of the aptamer-based COVID-19 assay using a glucometer in Fig. 1. The conformation-switching property of an aptamer in the target's presence is the basic principle for this displacement assay. Thus, the hybridization site for the complementary oligonucleotides on the aptamers was strategically selected based on their secondary structure, as predicted by M-fold [9]. The complementary antisense oligo-binding site on the aptamers and the release of antisense from the complex were initially confirmed through PCR, as depicted in Fig. 2. The conjugation of the complementary antisense DNA with the invertase enzyme through sulfosuccinimidyl-4-(N-maleimidomethyl) cyclohexane-1-carboxylate (sulfo-SMCC) linker was performed and confirmed with an electrophoretic mobility shift assay (EMSA), as shown in Fig. 3. The custom glucose sensor's design and layer-by-layer assembly monitored by cyclic voltammetry measurements are shown in Fig. 4. Optimization of invertase enzyme activity for reducing the assay time was conducted and shown in Figs. 5 and 6. An appropriate aptamer/antisense-invertase density over the MB surface is required for good analytical performance. Thus, optimization of the aptamer-antisense complex density on the MBs was performed, as shown in Fig. 7. A study was performed to identify the optimum time for the aptamer-target interaction and enzymatic (invertase) reaction, as shown in Fig. 8. Proof of aptamer displacement for detecting SARS-CoV-2 antigens with a custom glucose sensor is depicted in Fig. 9. Clinical sample testing for detection of SARS-CoV-2 with a glucometer is shown in Fig. 10. The tradeoff between the assay's sensitivity and specificity is shown by the receiver operator characteristic (ROC) curve in Fig. 11. The N protein concentration from SARS-CoV-2 clinical samples was estimated based on the proposed assay, shown in Fig. 12. The dataset used for these graphs is available in the Mendeley database.

The full-length DNA sequence of the aptamers for SARS-CoV-2 S and N protein and the corresponding complementary antisense strand, with their chemical modifications used in the assay, is presented in Table 1. The composition of various buffers that were used in the assay is listed in Table 2. An estimate of the test cost based on the cost of goods used in the assay is shown in Table 3. Clinical and demographic information of 8 healthy volunteers and 16 SARS-CoV-2 confirmed positive are summarized in Table 4. The primer sequences used in PCR amplification for initial confirmation of the antisense release from the respective aptamers upon target binding are shown in Table 5. The dataset used for these graphs is available in the Mendeley database.

**Table 1:** Aptamer and antisense sequences.

Aptamer Target	Aptamer Sequence (5′-3′)	Antisense Sequence (5′-3′)
SARS-CoV-2 N	Biotin/TTTTTTGCAATGGTACGGTACTTCCGGATGCGGAAACTGGCTAATTGGTGAGGCTGGGGCGGTCGTGCAGCAAAAGTGCACGCTACTTTGCTAA	Thiol/TTTTTTTTTTTTGACCGCCCCAGCCT
SARS-CoV-2 S	Biotin/TTTTTTCAGCACCGACCTTGTGCTTTGGGAGTGCTGGTCCAAGGGCGTTAATGGACA	Thiol/TTTTTTTTTTTTTGT CCATTAACGCCC

**Table 2:** Buffer composition used for various reactions.

Name	Composition
Washing and binding buffer	10 mM tris, 0.5 M NaCl, 1 mM EDTA, pH 7.4
Sucrose buffer (4×)	0.4 M citrate, 4 M sucrose, 20 mM CaCl_2_, 4 mM MgCl_2_, 2 mM EDTA, 11.1 mM glucose, pH 5.0
Measurement buffer (2×)	2 M sucrose, 2 mM ferrocene, 10 mM CaCl_2_, 2 mM MgCl_2_, 1mM EDTA in PBS pH 7.4

**Table 3:** List of reagents and cost of goods per SARS-CoV-2 test. Calculated cost based on MSRP listed on vendor websites. Cost of general laboratory consumables (*e.g.*, pipette tips) and instrumentation is not included.

Vendor	Item	Catalog #	Price (USD)
Sigma Millipore	Microcentrifuge tubes	T6649-500EA	0.10
Amazon	Glucometer test strip	Accu-Chek GuideMe	0.25
Sigma Millipore	Dynabeads magnetic beads	60210	2.20
Sigma	Invertase (*S. cerevisiae*)	I4504-5G	0.10
Integrated DNA Technology	SARS-CoV-2 N or S aptamer, antisense oligo	278779587, 278779588	0.50
Thermo Fisher	10% BSA solution	37525	0.05
Sigma	Dulbecco PBS buffer	D8537	0.01
Sigma	Citrate buffer	P4809	0.01
Sigma	Magnesium	M8266	0.001
Sigma	EDTA	E6758	0.002
Sigma	Calcium	21049	0.005
Sigma	Sucrose	S7903	0.01
Thermo Fisher	TCEP bond breaker	77720	0.005
Sigma	Sulfo-SMCC	573115	0.10

**Total cost per test**			**$3.20**

**Table 4:** Demographic information, symptoms, and measurement data for the cohort. SARS-CoV-2 positive subjects are “Patients,” while negative subjects are “Volunteers.” All glucose values are averages from independent triplicate measurements. Symptoms and comorbidities were all self-reported. Ages rounded to the nearest 10 years for confidentiality.

ID	Age (approx.)	Sex	Days between first positive test to sample	Days between symptom onset and sample	Fever	Cough	Fatigue	Shortness of breath	Chills	Anosmia	Ageusia	Comorbidities	Hospitalized?	Ct Value	Background Glucose [mg/dL]	ΔGlucose [mg/dL]
Patient 15	50	M	2	4	Yes	Mild	No	No	Yes	No	Yes		No	25.4	54.0	301.0
Patient 18	30	F	7	9	Yes	N/A	N/A	N/A	N/A	N/A	N/A		No	34.7	91.0	404.0
Patient 23	30	F	6	10	Yes	Mild	No	No	Yes	No	No		No	20.9	39.5	262.5
Patient 30	30	F	4	3	No	No	No	No	No	Yes	Yes	Pregnant	No	23.1	42.5	199.5
Patient 38	30	M	2	3	Yes	Mild	No	No	No	Yes	Yes		No	24.1	307.0	145.3
Patient 40	30	M	4	4	No	Mild	Mild	No	No	Yes	Yes	Asthma	No	28.6	51.0	68.3
Patient 42	40	M	5	7	Yes	No	Mild	No	Yes	Yes	Yes		No	22.5	58.0	217.3
Patient 56	20	F	5	7	No	No	No	No	No	Yes	Yes		No	27.2	63.5	328.2
Patient 57	30	F	4	6	Yes	No	No	No	No	No	No		No	26.0	93.0	359.3
Patient 61	20	F	9	10	No	No	No	No	No	No	No		No	31.2	58.5	187.2
Patient 63	20	M	7	15	Yes	Mild	No	No	No	Yes	Yes		No	32.2	181.5	334.2
Patient 68	30	F	4	8	No	Mild	Mild	Moderate	No	Yes	Yes	Asthma	No	33.1	47.0	87.0
Patient 72	30	F	3	8	Yes	No	Mild	No	No	Yes	No		No	21.7	43.0	122.3
Patient 74	20	M	5	6	Yes	No	No	No	Yes	No	No		No	29.5	50.0	136.3
Patient 77	60	M	6	5	Yes	Mild	Mild	No	No	Yes	Yes		No	23.4	55.0	201.0
Patient 78	30	M	3	6	No	Mild	No	No	No	No	No		No	30.3	57.5	130.2
Volunteer 1	40	M	N/A	N/A	No	No	No	No	No	No	No		No	N/A	40.0	27.7
Volunteer 2	30	M	N/A	N/A	No	No	No	No	No	No	No	Asthma	No	N/A	35.0	30.0
Volunteer 3	30	M	N/A	N/A	No	No	No	No	No	No	No		No	N/A	32.0	37.7
Volunteer 4	30	F	N/A	N/A	No	No	No	No	No	No	No		No	N/A	34.0	17.7
Volunteer 5	40	M	N/A	N/A	No	No	No	No	No	No	No		No	N/A	33.0	23.7
Volunteer 6	40	F	N/A	N/A	No	No	No	No	No	No	No		No	N/A	39.3	14.0
Volunteer 7	20	M	N/A	N/A	No	No	No	No	No	No	No		No	N/A	40.0	18.0
Volunteer 8	30	M	N/A	N/A	No	No	No	No	No	No	No		No	N/A	42.0	25.3

**Table 5:** PCR primers.

Aptamer Target	Forward Primer (5′-3′)	Antisense Sequence (5′-3′)
SARS-CoV-2 N	GCAATGGTACGGTAC	GACCGCCCCAGCCT
SARS-CoV-2 S	CAGCACCGACCTTG	TGTCCATTAACGCCC

## 2 Experimental Design, Materials, and Methods

**Fig. 13:**
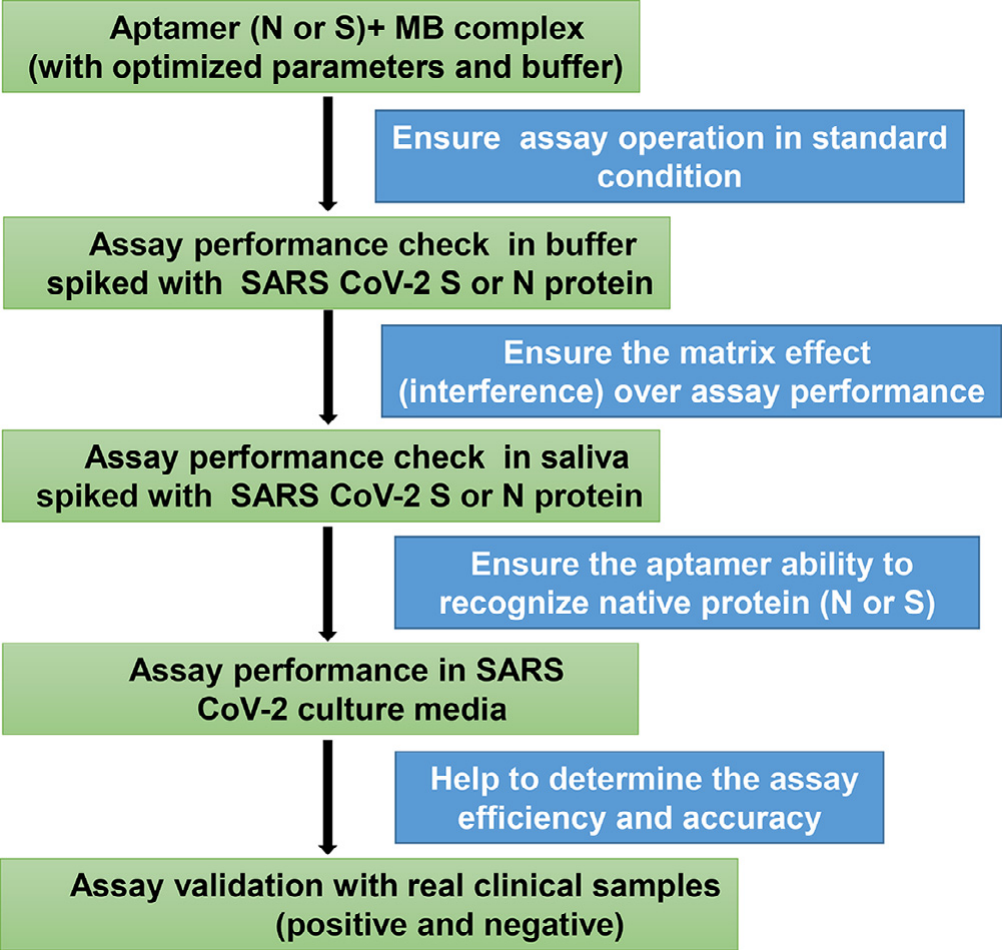
*Workflow.* The sequence of experiments to observe the assay output in the presence of different matrices.

We designed aptamers against SARS-CoV-2 S and N proteins [7,8] that were tagged with biotin, as well as complementary antisense sequences that were thiolated (Table 1). The aptamers and antisense oligonucleotides were ordered from Integrated DNA Technology (IDT). The following analytical-grade reagents were purchased from Thermo Fisher: bovine serum albumin (BSA, 10%); dithiobis(succinimidyl propionate (DSP); Dynabeads M-280 (2.8 µm) coated in streptavidin; tris(2-carboxyethyl) phosphine (TCEP). The following analytical-grade reagents were purchased from Sigma Aldrich: 4-(N-Maleimidomethyl) cyclohexane-1-carboxylic acid 3-sulfo-N-hydroxysuccinimide ester sodium salt (sulfo-SMCC); calcium chloride (CaCl_2_); citrate buffer; Dulbecco's potassium phosphate buffer (DPBS) with calcium and magnesium; ethylenediaminetetraacetic acid (EDTA); glucose, glucose oxidase type-VII from *Aspergillus niger*; invertase (Grade VII) from *Saccharomyces cerevisiae*; magnesium chloride (MgCl_2_); sodium borohydride (NaBH_4_); sucrose. Washing and binding buffer, sucrose buffer (4×), and measurement buffer (2×) were made for various reactions (Table 2). The following viral proteins were purchased from Sino Biological: SARS-CoV-2 Spike (S) and Nucleocapsid (N) proteins. We also obtained the following required reagents, media, and consumables: antibiotics (10,000 U/mL Penicillin-Streptomycin [Gibco]); culture media (Dulbecco's Modified Eagle's Medium/DMEM [Corning]); filters with various pore sizes (3, 10, 100 kDa, Amicon® [Millipore]). The reagents and goods needed to perform the SARS-CoV-2 detection assay and their cost per reaction are provided in Table 3. An off-the-shelf glucometer (Accu-Chek Guide Me) was used for assay validation in this study. Experiments were performed sequentially to verify and assess the assay performance, as shown in Fig. 13.

### 2.1 Conjugation of invertase with the antisense oligomers

Thiolated antisense oligomer strands were covalently conjugated to the invertase enzyme using a previously reported protocol [6]. Briefly, 6 µL of 0.5 M TECP was added to 30 µL of 1 mM thiolated antisense oligomer and incubated at room temperature (RT) for 2 h. Following this, the antisense strand was purified using a 3 kDa cutoff centrifugation filter. Next, 400 µL invertase was mixed with 1 mg of water-soluble sulfo-SMCC and the mixture was incubated for 2 h at RT on a shaker. The unbound sulfo-SMCC was removed by centrifugation with a 10 kDa cutoff filter following this incubation. The purified sulfo-SMCC linked invertase (sulfo-SMCC-invertase) was then mixed with the purified, reduced, thiolated antisense strand and kept on a shaker for 48 h at RT. Antisense oligomers that remained unconjugated were removed by using a 10  kDa cutoff filter, and the purified antisense-invertase conjugate was stored at 4 °C.

### 2.2 Hybridization of aptamer with the antisense-invertase conjugate

The conjugated invertase-antisense oligonucleotides were re-folded by heating for 10 min at 40 °C, and the biotin-tagged S protein and N protein aptamers were re-folded by heating for 3 min at 80 °C, followed by 5 min cooling at RT. Next, the antisense-invertase conjugate and aptamers were combined in the following manner: 20 µL of the invertase-antisense oligomer and 10 µL (0.5 mM) of the S or N aptamers were added to 170 µL DPBS and incubated at RT for 2 h to hybridize the aptamers with the invertase-oligomer. Following this, any unhybridized aptamers or invertase-oligomers were removed from the mixture by centrifugation with a 100 kDa filter. The purified aptamer/antisense-invertase hybridized complex was aliquoted and stored at 4 °C for further use.

### 2.3 Conjugation of aptamer/antisense-invertase complex and magnetic beads

The supernatant from the 200 µL of streptavidin-coated magnetic beads (MBs) was first discarded after placing the tube on a magnetic rack and then replaced with 600 µL of washing and binding buffer (Table 2). The MBs were then equilibrated in DPBS buffer and incubated with 200 µL of the biotinylated aptamer/antisense-invertase complex for 1 h at RT on a shaker. Any unbound aptamer/antisense-invertase complex was washed off with buffer, and the process was repeated 3×. To prevent biofouling, the resulting aptamer/antisense-invertase magnetic bead complex (MBC) was incubated in DPBS containing 1% BSA for 30 min at RT; after this incubation, the solution was discarded. The MBC was then resuspended in 400 µL of DPBS, and 50 µL aliquots (∼200 µg) were stored at 4 °C for subsequent assay.

### 2.4 Fabrication of the custom electrochemical glucose sensor

We created a custom electrochemical glucose sensor with a glass slide containing an evaporated gold electrode (5 nm Ti / 50 nm Au). The glass slide was chemically cleaned by submerging it in piranha solution (3:1 H_2_SO_4_:H_2_O_2_) for 1 min, washing it with milli-Q water, sonicating it in acetone and isopropanol for 5 min, and then rewashing it with milli-Q water. Next, the glass slide was electrochemically cleaned by submerging it in 0.5 M H_2_SO_4_ and sweeping the potential from -0.5 to +1.2 V, washing it with milli-Q water, and letting it air dry. The electrode was submerged in a DSP-NaBH_4_ solution (1 mL of 2 mg/mL DSP and 5 µL of 10 mg/mL NaBH_4_) and incubated at RT for 2 h to create a surface assembled monolayer (SAM). Following incubation, the electrode was washed sequentially with acetone, methanol, isopropanol, and milli-Q water, and allowed to air dry. The electrode was then submerged in 5 µM glucose oxidase and incubated overnight at 4 °C to link the SAM covalently. The next day, PBS was used to wash the electrode, which removed any unbound Glucose Oxidase (GOx). The electrode was then submerged in 1% ethanolamine and incubated at RT for 15 min, followed by a 10 min incubation in 1% BSA. We monitored the layer-by-layer assembly of the electrochemical glucose sensor using a CHI-760E electrochemical workstation with a 3-electrode configuration. We measured voltammograms with 1 mM ferrocene in 0.25 M KCl and 1× PBS from -0.5 to +0.8 V with a 50 mV/s scan rate. The electrochemical glucose sensor was stored at 4 °C.

### 2.5 Custom-made glucose sensor SARS-CoV-2 assay

Aliquots of 50 µL (200 µg) MBC (containing either N or S aptamers) in 1.5 mL microcentrifuge tubes (described in “Conjugation of aptamer/antisense-invertase complex and magnetic beads” section above) were allowed to equilibrate at RT. After equilibrating, 100 µL DPBS buffer spiked with SARS-CoV-2 N or S protein (matched with MBC) was added to the tubes and incubated at RT for 30 min with gentle agitation. Next, a magnet was used to concentrate/sequester the MBC, and 90 µL of the supernatant was transferred to another microcentrifuge tube containing 100 µL Measurement buffer (2×) (Table 2). The solution was incubated at RT for 40 min, after which 200 µL was transferred to the glucose sensor, and the result was reported using the CHI-760E electrochemical workstation (as described in “Fabrication of the custom electrochemical glucose sensor” section above).

### 2.6 Collection of nasopharyngeal swab and saliva samples

Nasopharyngeal swab (NPS) and saliva samples were collected from asymptomatic and symptomatic COVID-19 patients under institutional review board (IRB) approval (UCSD protocol #200477). NPS samples were collected in either DNA/RNA Shield storage medium (Zymo) or viral transport medium, which was prepared according to CDC guidelines [11]. Healthy volunteers also provided 300–500 µL saliva in sterile tubes using a passive drooling method [12]. Saliva samples contained no additives, and all clinical samples were stored in 300 µL aliquots at -80 °C. All samples were confirmed positive under clinical conditions via RT-qPCR tests: briefly, viral RNA was extracted from samples using the MagMax Viral/Pathogen Nucleic Acid Isolation Kit (Thermo), and RNA was amplified/quantified using the TaqPath COVID-19 multiplex RT-qPCR assay (Thermo). Saliva samples were blinded and tested in a Biosafety Level 3 (BSL-3) laboratory, and all experimentation followed UCSD Human Research Protections Program (HRPP) guidelines and regulations. Table 4 lists relevant demographic information for this cohort.

### 2.7 Glucometer-based SARS-CoV-2 assay

We used contrived and saliva samples from SARS-CoV-2 patients and healthy volunteers to test the glucometer-based SARS-CoV-2 assay. For samples used to detect N protein, 1% Triton was added to release the N protein into the medium, but no detergent was added to the samples used to detect S protein. For each sample type (contrived or saliva), 100 µL sample volumes were diluted with 100 µL DPBS buffer, after which half the diluted sample (100 µL) was added to 1.5 mL microcentrifuge tubes containing 200 µg MBC (complexed to N or S aptamers) and incubated at RT for 30 min with gentle agitation. After incubation, the MBC was concentrated/sequestered with a magnet, and 90 µL of the supernatant was transferred to new 1.5 mL microcentrifuge tubes containing 20 µL sucrose buffer (Table 2) and mixed. The second half of the diluted sample was not processed with MBCs but was directly added to a new 1.5 mL microcentrifuge tube containing 20 µL sucrose buffer to act as a background control. Both tubes (MBC-treated and background control) from each sample were then incubated in a water bath at 60 °C for one hour. Following incubation, 10 µL from each tube was transferred to a glucometer test strip, and results were displayed on a glucometer. The difference between the MBC-treated test and the background control test was calculated for each sample, and all measurements were repeated in triplicate.

### 2.8 Safety

Piranha solution is highly corrosive and should be handled with extreme precaution. All experiments with SARS-CoV-2 samples were performed in the UC San Diego Division of Infectious Diseases Biosafety Level 3 (BSL-3) laboratory following the oversight of the UC San Diego Institutional Biosafety Committee (IBC).

### 2.9 Statistical analysis

Data were collected from at least three independent, biological replicates. Error bars on figures are equal to ±1 standard deviation (SD). All statistical analyses were performed with Origin 9.0 or MATLAB. Limit of detection (LOD) analyses were calculated as described previously where LOD = 3×SD of slope [13].

## Ethics Statement

Ethical approval of the study was obtained from Institute review board (IRB) at University of California, San Diego (UCSD protocol #200477) and informed consent was taken from all subjects.

## CRediT Author Statement

**Naveen K. Singh:** Conceptualization, Investigation, analysis, writing; **Partha Ray:** Conceptualization, Investigation, analysis, writing; **Aaron F. Carlin:** Investigation; **Celestine Magallanes:** Resources; **Sydney C. Morgan:** Resources, writing; **Louise C. Laurent:** Resources, Supervision; **Eliah S. Aronoff-Spencer:** Conceptualization, Formal analysis, Supervision, Project administration, Funding acquisition; **Drew A. Hall:** Conceptualization, Formal analysis, Writing – original draft, Writing – review & editing, Supervision, Project administration, Funding acquisition.

## Declaration of Competing Interest

The authors declare that they have no known competing financial interests.
